# La lèpre de l’enfant à Thiès/Sénégal: signal d’une recrudescence?

**DOI:** 10.11604/pamj.2017.27.174.12150

**Published:** 2017-07-05

**Authors:** Pauline Dioussé, Haby Dione, Mariama Bammo, Ndiaga Gueye, Thierno Abdoul Aziz Diallo, Fatou Seck, Ramatoulaye Diagne Gueye, Mame Thierno Dieng, Fatma Sarr Fall, Moustapha Diop, Bernard Marcel Diop, Mamadou Mourtalla Ka

**Affiliations:** 1 UFR des sciences de la Santé, Université de Thiès, Sénégal; 2 Service de Dermatologie-Vénérologie, Hôpital Régional de Thiès, Sénégal; 3 Dermatologie, Université Cheikh Anta Diop, Dakar, Sénégal; 4 Centre de Santé de Mbour, Région Médicale de Thiès, Sénégal; 5 Centre de Santé de Thiès, Région Médicale de Thiès, Sénégal

**Keywords:** Lèpre, enfant, maladie de Hansen, Sénégal, Leprosy, child, Hansen’s disease, Senegal

## Abstract

La lèpre est une maladie infectieuse, transmissible. Le nombre de nouveaux cas de lèpre chez l’enfant au Sénégal connait une légère hausse depuis 2013 selon l’OMS. Les objectifs du travail étaient d’étudier les aspects épidémiologiques, cliniques, thérapeutiques et évolutifs de la lèpre de l’enfant dans les zones géographiques de deux villages de reclassement de la région de Thiès. Il s’agit d’une étude rétrospective menée sur une période de 3 ans (2013-2015). Etaient inclus tous les nouveaux cas de maladie de Hansen âgés de 0 à 15 ans. En trois ans, 39 enfants étaient inclus, avec une prédominance de garçons (n=23, 59%). Parmi ces enfants, 27 (66,7%) provenaient d’un village de reclassement social des lépreux. Il existait une atteinte d’un membre de la famille dans 27 cas (69,2%). Plus de la moitié des enfants, soit 23 cas (58,9%) avaient une lèpre multi bacillaire (Lépromateuse-Lépromateuse). Tous les enfants étaient mis sous traitement durant 12 mois, au terme desquels trente-six (92,3%) enfants étaient guéris. La lèpre est encore présente au Sénégal malgré les efforts du programme national de lutte. Au regard de ces résultats, il est important de souligner l'importance de la stratégie de dépistage actif ciblé sur les enfants, qui semble avoir montré son efficacité dans la région. La détection précoce, la recherche des contacts et le traitement précoce sont autant de facteurs importants dans la réduction de la contagiosité de la lèpre.

## Abstract (en)

Leprosy is an infectious and transmissible disease. According to the WHO, the number of new cases of leprosy in children in Senegal has risen moderately since 2013. This study aimed to analyze the epidemiological, clinical, therapeutic and evolutionary features of leprosy in children in the geographical areas of two social rehabilitation villages in the region of Thiès. We conducted a retrospective study over a period of 3 years (2013-2015). All new cases of Hansen’s disease aged 0 -15 years were included. Over the three year period, 39 children were included in the study, with a boy predominance (n=23, 59%). Among these children, 27 (66.7%) came from a social rehabilitation village for leprosy patients. One family member was affected by leprosy in 27 cases (69.2%). More than half of the children (23 cases, 58.9%) had multibacillary leprosy (lepromatous-lepromatous). All children underwent a 12-month treatment, at the end of which thirty-six (92.3%) children were healed. Leprosy is still present in Senegal despite the efforts made by the national programme to combat leprosy. In the light of these results, it is important to emphasize the role of active screening strategy targeted to children, which seems to have shown its effectiveness in the region. Early detection, contact tracing and early treatment are important factors in the reduction of the contagiousity of leprosy.

## Introduction

La lèpre est une maladie infectieuse, transmissible, due à Mycobacterium leprae, ayant comporté 215 656 nouveaux cas dans le monde en 2013 [1]. Bien que la lèpre ne constitue pas un problème de santé publique au Sénégal depuis 1995, le nombre de nouveaux cas de lèpre chez l’enfant au Sénégal a connu selon l’OMS une légère hausse en 2013 (40 contre 33 en 2012) [1]. Parmi les 9 villages de reclassement social des lépreux, créés en 1978 au Sénégal, la région de Thiès en abrite deux: Touba Peykouck et Mballing [2]. Le but de ce travail était de calculer l’incidence de la lèpre chez les enfants vivants dans ces zones géographiques et d’en étudier les aspects épidémiologiques, cliniques, thérapeutiques et évolutifs.

## Méthodes

**Figure 1:**
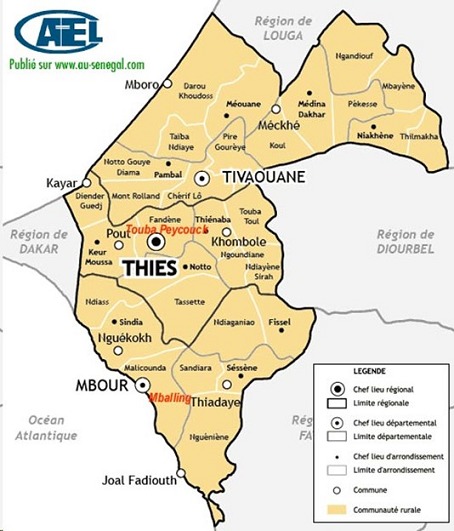
La carte de la région de Thiès indiquant les deux villages de reclassement

La région de Thiès est constituée de 3 départements: Thiès, Mbour et Tivaouane. Sa superficie est de 6600 km^2^et sa population est estimée à 1 788 864 habitants pour une densité de 271 habitants/km^2^. La ville de Thiès est située à 70 kilomètres de Dakar la capitale [3]. Il s’agissait d’une étude rétrospective dans le centre de santé du département de Thiès (couvrant l’aire géographique de Touba Peykouck) et dans celui du département de Mbour (couvrant Mballing) (Figure 1). Elle était menée du 1er janvier 2013 au 31 décembre 2015. Etaient inclus tous les nouveaux patients âgés de 0 à 15 ans, enregistrés dans ces services pour une maladie de Hansen clinique. Etaient non inclus, les rechutes et les retraitements avant 2013. Les données provenaient des registres de suivi et de traitement. Elles concernaient les aspects: sociodémographiques: âge, sexe, provenance d’un village de reclassement ou non, atteinte d’un membre de la famille; Cliniques selon la classification de Riedley et Jopling [4] et les critères d´invalidité de l´Organisation mondiale de la santé (OMS) (Tableau 1); Thérapeutiques: observance, irrégularité du traitement, perdu de vue, abandon, retraitements (traitement est repris car mal adapté par rapport à la forme clinique), rechutes (traitement repris pour reprise évolutive des lésions malgré un traitement correctement conduit). Les modalités du traitement recommandées par l’OMS dans les zones d’endémies étaient:

**Tableau 1:** La classification de Riedley et Jopling et les critères d'invalidité de l'OMS

Topographie	Types d’invalidité	Degré
Pieds et mains	Absence d'anesthésie, pas de déformation ni de lésion visible,	0
	Anesthésie, mais pas de déformation ou de lésion visible,	1
	Présence d'une déformation ou d'une lésion visible	2
Yeux	Absence de problèmes oculaires imputables à la lèpre, aucun signe de perte d'acuité visuelle,	0
	Présence de problèmes oculaires imputables à la lèpre, mais aucune baisse corrélative d'acuité visuelle (au moins égale à 6/60),	1
	forte baisse de l'acuité visuelle (acuité inférieure à 6/60).	2

**Multibacillaires**: La rifampicine (RMP): 10 mg/kg/mois, supervisée (Enfant de moins de 10 ans : 300 mg; de 10 à 14 ans: 450 mg); La diamino-diphényl sulfone (DDS): 1,5 mg/kg/j (Enfant de moins de 10 ans: 25 mg; de 10 à 14 ans: 50 mg); La clofazimine (CLO): 1 mg/kg/jour (Enfant de moins de 10 ans: 50 mg 2 fois par semaine; 10 à 14 ans: 50 mg tous les 2 jours) et en prise supervisée 3 mg/kg/mois (Enfant de moins de 10 ans: 100 mg, de 10 à 14 ans: 150 mg).

Durée du traitement: 12 mois.

**Paucibacillaires**: RMP: 10 mg/kg/mois, supervisée; DDS: 1,5 mg/kg/j

Durée du traitement: 6 mois

**Modalités de suivi**: L’OMS préconise la PCT accompagnée (PCT-A) basée sur la remise, dès que le diagnostic est posé, de l’ensemble des plaquettes de PCT (6 à 12 mois selon la forme de lèpre) après s’être assuré qu’un accompagnement du malade par un membre de sa famille ou de son entourage est réalisé.

**Critères de guérison**: Critères cliniques avec nette régression des lésions cutanées durant le traitement.

**Taux d’achèvement du traitement**: Paucibacillaires (durée 6 mois): le patient devait prendre 6 doses en 6 à 9 mois maximum; Multibacillaires (durée 12 mois): le patient devait prendre 12 doses en 12 à 18 mois maximum.

**Evolutives**: Mutilations, guérison.

L’analyse statistique des données était faite à partir du logiciel EPI Info version 3.5.4. (CDC Atlanta). Pour comparer les proportions, le test du Chi Carré était utilisé avec un seuil de significativité p < 0,05.

## Résultats

**Figure 2:**
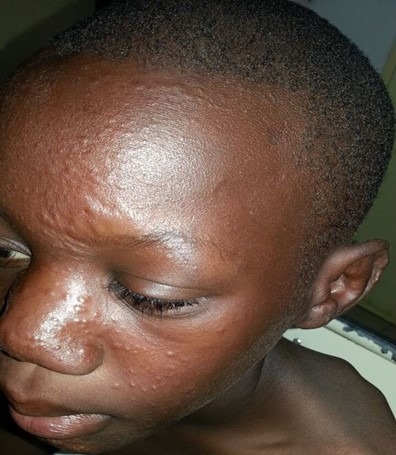
Les lésions papuleuses infiltrées du visage, des oreilles chez un enfant atteint de lèpre

Sur une cohorte de 63 patients atteints de lèpre recensés dans la région, 39 (61,9%) étaient des enfants âgés de moins de 15 ans dont 27 (69,2%) provenaient du centre de traitement de Mbour et 12 (30,8%) de celui de Thiès. Le nombre de patients était de 9 (23,1%) en 2013, 20 (51,3%) en 2014 et 10 (25,6%) en 2015. L’âge moyen était de 9,7 ans (extrêmes: 3-15). Le sexe ratio Homme/Femme était de 1/6. Parmi ces enfants, 27 (66,7%) provenaient d’un village de reclassement social des lépreux et 13 (33,3%) des quartiers environnants. Un membre de la famille était atteint dans 27 cas (69,2%). Plus de la moitié des enfants avaient une lèpre multi bacillaire (LL) 23 (58,9%) (Tableau 2, Figure 2). Selon les critères d´invalidité de l´OMS, 3 enfants avaient une atteinte des pieds et des mains de degré 1 (anesthésie sans déformation ni lésion visible). Sur le plan thérapeutique, tous les enfants étaient mis sous traitement durant 12 mois, avec une bonne observance dans 36 cas (92,3%). Trente-six (92,3%) enfants étaient guéris, deux enfants étaient en reprise de traitement et un enfant avait rechuté.

**Tableau 2:** La répartition des enfants selon la forme Clinique

Formes cliniques	Nombre	Pourcentage (%)
Indéterminé (I)	3	7,7
Borderline-tuberculoïde (BT)	13	33,3
Borderline-lépromateuse (BL)	5	12,8
Lépromateuse-lépromateuse (LL)	18	46,2

## Discussion

Notre étude a mis en évidence une proportion élevée d’enfants et de formes multi- bacillaires, potentiellement contagieuses. Cette constatation traduit un retard diagnostic et le maintien d’une transmission active de l’infection dans la communauté. Dans notre série, les enfants de 0 à 15 ans représentant 61,9% du total des cas de lèpre enregistrés dans la région, apparaissent comme le groupe le plus vulnérable. Ce taux est largement supérieur à celui trouvé à Dakar en 2011 (12%) [5]. La prédominance pédiatrique trouvée dans notre série est probablement due au fait qu’en 2014, une organisation non gouvernementale allemande DAHW (Deutsche Lepra- und Tuberkulosehilfe e.V: German Leprosy and Tuberculosis Relief Association), dans le cadre de la promotion des activités d’élimination de la lèpre au sein des communautés, a mis en place une stratégie de dépistage des enfants atteints. Cette stratégie consiste à la formation des enseignants des environs du village de reclassement de Mballing à la reconnaissance des lésions cutanées. Cette stratégie a permis de diagnostiquer un nombre important d’enfants durant l’année 2014. Dans notre série, la moyenne d’âge des enfants 9,7 ans est moins élevée que dans l’étude de Singal et al. en Inde [6]. Cette différence pourrait s’expliquer par le dépistage actif des enfants de notre étude par rapport à l’étude indienne. Quant à la prédominance masculine trouvée ici, elle ne reçoit pas d’explication précise, bien que cette notion soit classique.

Les villages de reclassement social des lépreux ont été mis en place au Sénégal par décret n°65-128 du 04 mars 1965 portant l’organisation des villages de lépreux, et par la loi 76-03 du 25 mars 1976 relative au traitement de la lèpre et au reclassement social des lépreux guéris et mutilés [2]. Ainsi neuf villages éparpillés sur l’ensemble du territoire sénégalais ont été créés. Dans la région de Thiès, deux villages de reclassement ont été implantés: Mballing dans le département de Mbour et Touba Peykouck dans celui de Thiès [2]. Avec l’urbanisation galopante, les villes ont rejoint et englouti ces villages de reclassement; les enfants fréquentant désormais les mêmes écoles et les mêmes aires de jeu. Actuellement, l’existence et le cadre légal de ces villages de reclassement social des lépreux guéris et mutilés doivent interpeller les décideurs. Ces derniers devraient réfléchir sur l’abrogation de la loi relative à la création de ces villages et mettre l’accent sur les facteurs de transmission. Cette dernière se fait essentiellement par des gouttelettes de Pflügge. La problématique actuelle est de rompre la chaine de transmission. Chez l’enfant, les formes paucibacillaires sont les plus fréquentes selon Sasidharanpilla et al [7]. Dans notre série, 23 enfants (58,9%) étaient classées multi bacillaires et 27 enfants (69,2%) avaient un contact étroit avec un membre de la famille atteint de lèpre. Ces facteurs pourraient contribuer à la propagation de la maladie et il s’y ajoute le fait que les lésions dermatologiques sont les signes les plus apparents, précoces, mais de valeur prédictive faible car non spécifique, souvent négligé par l’entourage [8]. Sur le plan thérapeutique, les recommandations de l'OMS étaient appliquées à tous les patients. La poly chimiothérapie (PCT) guérit la lèpre et diminue l’infectiosité dans la communauté. Dans l’étude de Seydi et al. au Sénégal, le taux de couverture en PCT était de 100%, le taux de guérison observé était de 89% et le taux de perdus de vue de 8% [9]. Dans notre série, trente-six (92,3%) enfants étaient guéris, deux enfants étaient en reprise de traitement et un enfant avait rechuté. Il faut cependant maintenir l’observation à plus long terme pour dépister la survenue et la propagation d’une résistance de la lèpre aux médicaments.

## Conclusion

La lèpre, maladie contagieuse, est encore présente au Sénégal malgré les efforts du programme national de lutte contre la lèpre. Au regard de ces résultats, il est important de souligner l´importance de la stratégie de dépistage actif ciblé sur les enfants, qui semble avoir montré son efficacité dans la région. La détection précoce, la recherche des contacts et le traitement sont autant de facteurs importants qui peuvent contribuer à réduire la contagiosité de la lèpre dans la communauté.

### Etat des connaissances actuelles sur le sujet

- La forme indéterminée est le mode de début de la maladie et s’observe le plus souvent chez l’enfant;
- Le taux de lèpre chez l’enfant est le reflet du niveau d’endémicité d’un pays;
- La transmission se fait par des gouttelettes de Pflügge.

### Contribution de notre étude à la connaissance

- Plus de la moitié des enfants avaient une lèpre multi bacillaire, donc contagieuse, et le nombre d’enfants dans la cohorte est supérieur à celui des adultes;
- La stratégie de la formation des enseignants d’un département à la reconnaissance des lésions cutanées a permis de dépister un nombre important d’enfants;
- La problématique de l’efficience des villages de reclassement se pose du fait de l’urbanisation galopante des villes africaines.

## Conflits d’intérêts

Les auteurs ne déclarent aucun conflit d'intérêt.
